# Different Manifestations of Persistent Sciatic Artery and Possible Treatment Options: A Series of Four Cases

**DOI:** 10.3390/diagnostics14212383

**Published:** 2024-10-25

**Authors:** Laura Maria Cacioppa, Marzia Rosati, Marco Macchini, Nicolo’ Rossini, Pietro Boscarato, Vincenzo Vento, Matteo Vocaturo, Andrea Coppola, Enrico Paci, Roberto Candelari, Chiara Floridi

**Affiliations:** 1 Department of Clinical, Special and Dental Sciences, University Politecnica delle Marche, 60126 Ancona, Italy; l.m.cacioppa@univpm.it (L.M.C.); c.floridi@staff.univpm.it (C.F.); 2 Division of Interventional Radiology, Department of Radiological Sciences, University Hospital “Azienda Ospedaliera Universitaria delle Marche”, 60126 Ancona, Italy; marzia.rosati@ospedaliriuniti.marche.it (M.R.); marco.macchini@ospedaliriuniti.marche.it (M.M.); pietro.boscarato@ospedaliriuniti.marche.it (P.B.); roberto.candelari@ospedaliriuniti.marche.it (R.C.); 3 Vascular Surgery Unit, Aortic Team, Lancisi Cardiovascular Center, 60100 Ancona, Italy; vincenzovento1987@gmail.com (V.V.); matteovoca@gmail.com (M.V.); 4 Diagnostic and Interventional Radiology Unit, Circolo Hospital, ASST Sette Laghi, 21100 Varese, Italy; andrea.coppola@asst-settelaghi.it; 5 Department of Medicine and Technological Innovation, Insubria University, 21100 Varese, Italy; 6 Unit of Radiology, IRCCS INRCA, 60127 Ancona, Italy; e.paci@inrca.it; 7 Division of Radiology, Department of Radiological Sciences, University Hospital “Azienda Ospedaliera Universitaria delle Marche”, 60126 Ancona, Italy

**Keywords:** persistent sciatic artery (PSA), endovascular treatment, arterial embolization, sciatic aneurysm, lower limb ischemia, peripheral arterial disease, intermittens claudication

## Abstract

Persistent sciatic artery (PSA) is a rare vascular anomaly classified into five types according to the Pillet and Gauffre classification system. Although PSA may be detected as an incidental finding, symptomatic cases account for approximately 80% of all cases and have variable clinical presentations. Due to the frequent ischemic and aneurysmal complications, PSAs can lead to limb-threatening conditions requiring prompt identification and adequate treatment management. In this paper, we present a series of four cases of PSA with extremely different anatomical characteristics, patients’ ages, medical histories and clinical presentations. All cases were diagnosed in our institution and managed after multidisciplinary discussions involving vascular surgeons and interventional radiologists. The series included three women and one man. In two cases, one of which included gluteal and back pain, pulsatile masses were found. Two patients had lower-limb chronic ischemia, one with rest pain and one with IIb claudication. Whereas selective angiography was performed only in endovascular approaches, computed tomography angiography (CTA) was performed on all patients as the decisive diagnostic modality. In our series, treatment strategies were selected on the basis of clinical and anatomical factors, and after the evaluation of the potential risks and benefits of each technique. Treatment was medical in two cases, endovascular in one case and hybrid in one case.

## 1. Introduction

**Figure 1:**
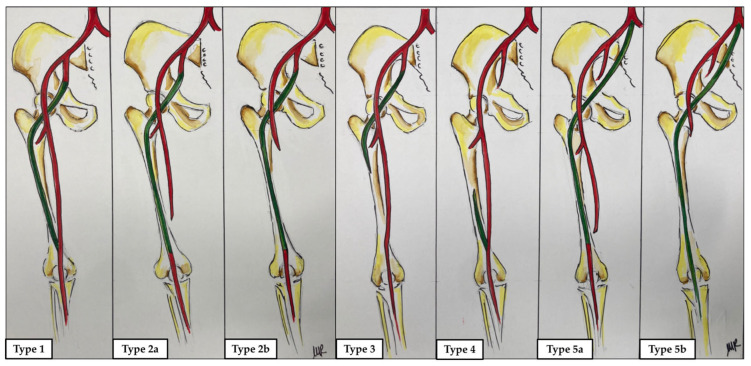
Persistent sciatic artery (PSA) classification system by Pillet (1980), modified by Gauffre (1994) [6,7]. Five types of persistent sciatic artery (colored in green) are described: type 1, a complete PSA runs along with a complete superficial femoral artery (SFA, colored in red); type 2, a complete sciatic artery runs along an incomplete (type 2a) or absent SFA (type 2b); type 3, only the proximal tract of a PSA persists with a normal SFA; type 4, only the distal tract sciatic artery persists with a normal SFA; type 5, the PSA originates from the median sacral artery and runs along either a developed (type 5a) or an underdeveloped SFA (type 5b).

Persistent sciatic artery (PSA) is a rare axial vascular malformation first described by P.H. Green in 1832 in a post-mortem case [1] and occurring in 0.025–0.040% of the population [2]. The sciatic artery (SA), a branch of the umbilical artery, is responsible for the embryonic blood supply to the lower limbs. Commonly, the distal part of the SA regresses during the third gestational month, when the external iliac artery develops, and lower limb perfusion is provided by the superficial femoral artery (SFA) [3]. However, when the SA fails to involute, it runs through the posterior thigh downward to the popliteal artery along the sciatic nerve [3,4]. The lack of regression of the SA may be related to regular development, hypoplasia or agenesis of the iliac–femoral axis [5]. According to anatomical characteristics, Pillet et al. classified PSA into four types, with a fifth type, where the PSA originates from the median sacral artery, later added by Gauffre [6,7]. The Pillet classification system (modified by Gauffre) is summarized in Figure 1. 

PSA has a variable clinical presentation. Despite PSAs detected as incidental findings being described, symptomatic cases account for ~80%, frequently due to sciatic neuralgia [8,9]. In 48% of cases, the repeated compressions and the inherent connective tissue defects in the primitive arterial wall of PSA result in the development of buttock aneurysm [10]. This can cause a painful pulsatile mass with compressive neuropathy, sudden rupture or ischemic manifestations due to thrombotic occlusion or distal embolism [11,12]. PSA can be long misdiagnosed as chronic obliterative arteriopathy or Buerger’s disease, with consequent treatment delays that may lead to amputation. Therefore, prompt identification using duplex ultrasound (DUS) and adequate diagnosis and treatment planning with computed tomography angiography (CTA) are needed [13,14]. Due to its potentially harmful complications and its high amputation risk, PSA is considered a highly morbid condition requiring prompt identification to prevent aneurysm rupture and distal embolism [9]. The treatment management of PSA depends on vascular anatomy (complete or incomplete PSA and complete or incomplete SFA), clinical symptoms and aneurysm formation [14]. In the review by Ahn-Min et al., a new classification system based on anatomical features was proposed with the aim of providing guidance for decision making and suggesting the optimal role of open, endovascular and hybrid treatment strategies [15]. PSA types without aneurysmal degeneration may be managed with optimal medical treatment and active surveillance [16,17]. Elective percutaneous transluminal angioplasty (PTA) with stenting or catheter-directed thrombolysis are also indicated for symptomatic cases with complete PSAs or SFAs [9]. In PSA with incomplete SFAs, the most common anomaly, an additional femoro-popliteal bypass with ligation of the proximal popliteal artery to the distal anastomosis may be needed [15]. PSA aneurysms with complete SFAs can be treated by endovascular coiling or stent-graft insertion while open surgical excision is increasingly abandoned due to the high risk of sciatic nerve damage [18,19,20]. Since no guidelines or threshold sizes have so far been reported, the decision regarding PSA aneurysm elective repair should be based on different anatomical and clinical factors [15,17]. The aneurysmal rupture risk, intraluminal thrombus characteristics, aneurysmal changes, bacterial or mycotic infective complications, pain and severe claudication should be considered [21,22]. In the present series, we report the experiences of our center in the management of PSA and its aneurysmal complications by describing four clinical scenarios with different treatment approaches.

## 2. Cases

### 2.1. Case 1 

**Figure 2:**
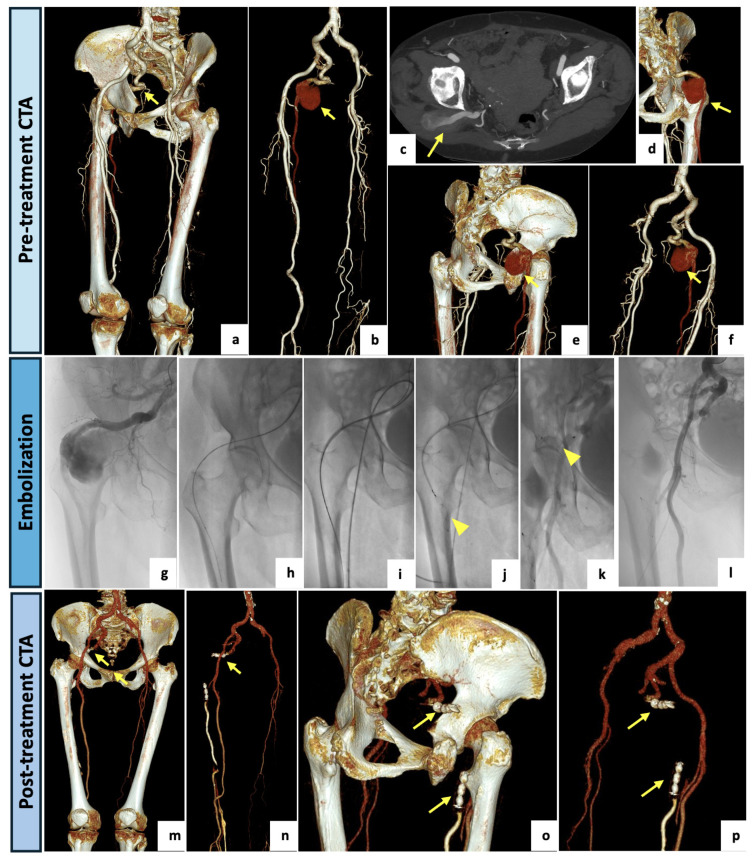
(**a**) VR reconstruction of the preoperative CTA showing a right PSA originating from the internal iliac artery (arrow), continuing as a popliteal artery and associated with a normal SFA (type 1). (**b**–**f**) Three-dimensional reconstructions, with and without bone structures, and axial maximal intensity projections showing a huge aneurysmal degeneration (57 mm in the AP caliber) of the PSA in the posterior aspect of the thigh (arrows). (**g**) Intraprocedural selective angiography of the right PSA performed after obtaining a left humeral access with a 4F-sheat (60 cm), confirming a large aneurysm. (**h**,**i**) Intraprocedural images obtained after right common femoral artery access with a 6F-sheat and right PSA selective catheterization. (**j**,**k**) Aneurysm exclusion with “sandwich technique”: embolization through two vascular plugs deployed in the outflow and in the inflow vessel (arrowheads). (**l**) At final angiography, a nearly complete occlusion of the aneurysm (small residual slow flow) was documented. (**m**–**p**) VR reconstruction of postoperative CTA (1-month), demonstrating the complete exclusion of the PSA aneurysm. Both inflow and outflow were occluded by vascular plugs (arrows).

A 79-year-old woman was admitted to our hospital for intense right flank and buttock pain with a burning sensation in the gluteal region radiating down the back of her right lower limb and preventing her from walking normally. She had no significant medical history other than experiencing several similar episodes of pain. Physical examination revealed stable vital signs, palpable and symmetric femoral pulses, normal distal bilateral perfusion, no claudication or ulcers and no clinical signs of superficial or deep vein thrombosis. Neurological examination revealed no motor or sensory deficit. Blood tests on admission were unremarkable. At DUS examination of the lower limbs, a normal right common femoral–external iliac axis was detected, with a regular right ankle–brachial index. The left-limb DUS showed a regular common femoral–external iliac axis with a hypoplasic SFA and popliteal low flow pattern. The ankle–brachial index was bilaterally normal. A CTA examination (Figure 2) showed a right PSA originating from the internal iliac artery, running through the greater sciatic foramen, entering in the thigh and continuing as a popliteal artery, supplying the distal arteries of the leg. The PSA was associated with a normal SFA (type 1). An aneurysmal degeneration of the PSA, measuring 57 mm in the maximal caliber with poor mural thrombus, was found in the posterior aspect of the thigh. On the left limb, a fully developed and completely occluded PSA with a hypoplastic SFA (type 2A) was detected. The popliteal artery was supplied by collateral pathways originating from the hypoplastic SFA and the deep femoral axis. Having confirmed that the patient was not suffering from left lower limb claudication interfering with normal activities, the case was collegially discussed and evaluated as eligible for endovascular management of the right aneurysmatic PSA. The procedure was performed in our institutional angiosuite (flat-panel C-arm system, Artis zee, Siemens Healthcare, Erlangen, Germany). A right common femoral artery access was performed under local anesthesia (10 mL of Mepivacaine 2% solution) and a 6F-sheat (45 cm) introducer sheath was positioned. A left humeral access with a long 4F-sheat (60 cm) was also utilized for diagnostic angiographies. The left femoral access was avoided due to a focal chronic dissection in the left external iliac artery, as shown by the CTA. The right PSA was selectively catheterized, and the aneurysm was embolized with the “sandwich technique” to avoid retrograde recanalization. Two vascular plugs (AVP—Abbott Medical, North Plymouth, MN, USA) were deployed in the outflow (12 mm) and in the inflow vessel (14 mm). The final angiograms showed a nearly complete occlusion of the aneurysm (small residual slow flow). The procedure was successfully completed without complications. The patient was discharged after two days with single antiplatelet therapy. At the clinical follow-up, a complete disappearance of symptoms and no further need for pain medication were confirmed, and the 1-month CTA follow-up examination showed the complete exclusion of the PSA aneurysm.

### 2.2. Case 2

**Figure 3:**
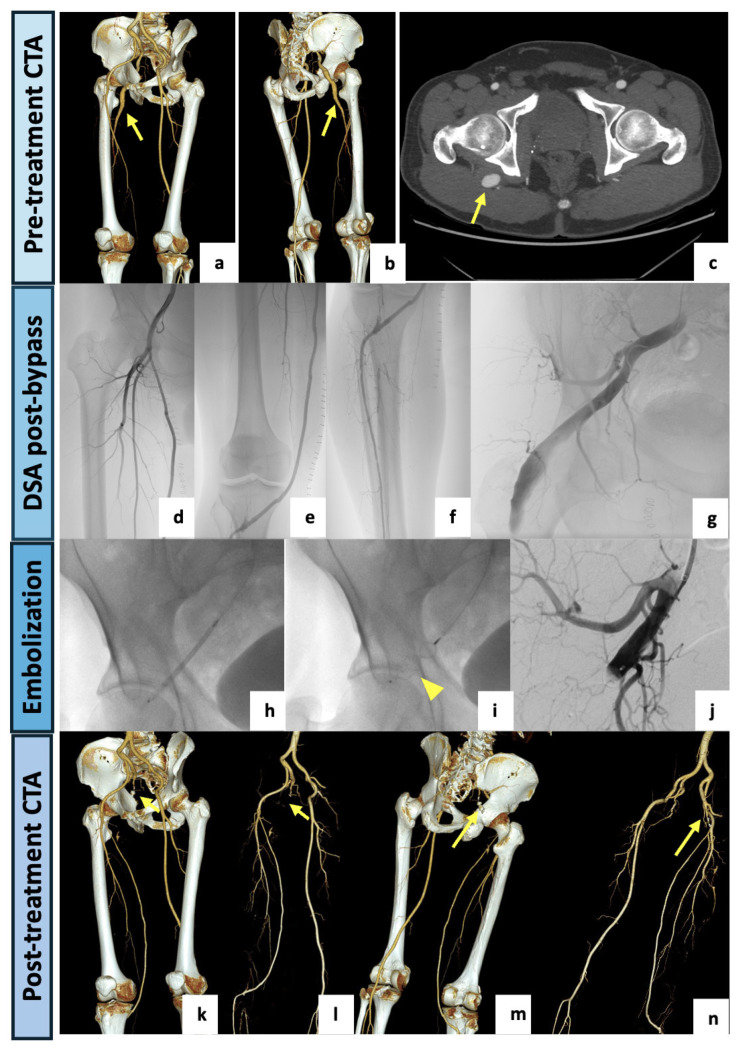
(**a**,**b**) VR reconstruction of the preoperative CTA showing a right complete PSA originating from the internal iliac artery (arrow). The right external iliac artery appears hypoplasic and the SFA appears incomplete and diminished in caliber (type 2a). (**c**) Axial view showing an aneurysmal dilatation (23 mm) of the PSA with luminal thrombus (arrow). A hybrid approach was preferred, with the first step consisting of a left femoro-tibial bypass with the anterior tibial as the target artery. (**d**–**f**) Selective angiographic images preliminary to the endovascular step showing a patent femoro-tibial bypass. (**g**–**j**) After a contralateral 6F-sheat femoral access, the aneurysmal PSA was selectively catheterized and completely embolized using a 12 mm Amplatzer Vascular Plug II (arrowhead) deployed immediately distally to the internal pudendal artery origin. (**k**–**n**) VR reconstructions of the postoperative CTA (3-month) demonstrating the patent femoro-tibial bypass with complete exclusion of the aneurysmal PSA (arrows).

The next patient was a healthy 53-year-old male referred to the Vascular Surgery Department of our center for critical ischemia with rest pain of the right lower limb. Upon physical examination, stable vital signs, palpable and symmetric femoral pulses, absent right popliteal and pedal pulses and mild hypothermia of the right foot were detected. Neurological examination revealed no motor or sensory deficit. The CTA examination (Figure 3) showed a right aneurysmatic complete PSA (23 mm in the maximal caliber) with extensive luminal thrombus originating from the internal iliac artery. The right external iliac artery was hypoplastic, probably due to a steal syndrome caused by the PSA. The ipsilateral SFA appeared incomplete and noticeably reduced in caliber (type 2a). A thrombotic occlusion of the left popliteal artery and left tibio-peroneal trunk tibial axis with filling thrombotic defects at the anterior tibial artery were observed. The posterior tibial and peroneal arteries were patent. The case was collegially discussed, and a hybrid approach was preferred. Firstly, a right femoro-tibial bypass with the anterior tibial as the target artery was performed under general anesthesia using the ipsilateral great saphenous vein in a reversed fashion. After two days, the patient subsequently underwent endovascular embolization of the aneurysmal PSA. The procedure was performed in our angiographic suite. A left femoral access was obtained under local anesthesia. The sciatic artery was catheterized and embolized using a 12 mm Amplatzer Vascular Plug II (Abbott Medical, North Plymouth, MN, USA) deployed immediately distally to the internal pudendal artery origin. Both of the procedures were conducted without complications. At the 3-month CTA follow-up examination, the bypass was patent, and the aneurysm was completely excluded.

### 2.3. Case 3

**Figure 4:**
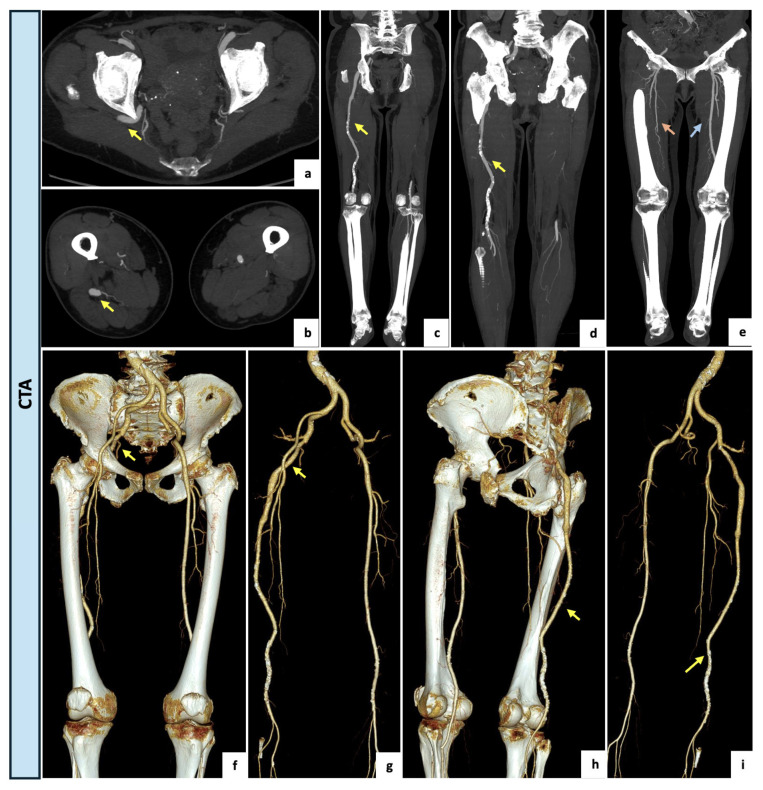
(**a**–**d**) Axial and coronal maximal intensity projection views of the CTA demonstrating a right complete and ectatic PSA of 13 mm (yellow arrow) originating from the internal iliac artery and accompanied by an incomplete ipsilateral SFA interrupted at Hunter’s canal (type 2a). (**e**) Coronal view of lower limbs with a right incomplete ipsilateral SFA (orange arrow) and a regular contralateral femoral axis (blue arrow) (**f**–**i**) Three-dimensional reconstructions showing the ectatic PSA (yellow arrow) running through the greater sciatic notch, following the proximal course of the sciatic nerve and continuing as popliteal–tibial axis.

An 81-year-old female with no major comorbidities and no history of peripheral vascular disease was referred to the Emergency Department of our hospital for the presence of moderate and recurrent back pain occasionally requiring pain medication. Upon examination, the patient exhibited an antalgic gait, normal vital signs, and regular perfusion, sensation and motor function of the lower extremities. A diminished right femoral pulse in combination with a regularly palpable popliteal pulse (named Cowie’s sign) was detected. The CTA examination (Figure 4) revealed the incidental finding of a right complete PSA originating from the internal iliac artery, with an incomplete ipsilateral SFA (type 2a). In this case, the PSA had developed as an ectatic arterial continuation (13 mm) of the internal iliac axis running through the greater sciatic notch, penetrating the bundles of the gluteus maximus muscle and following the proximal course of the sciatic nerve. Distally, the PSA had a regular course as popliteal–tibial axis with the high-origin anterior tibial artery. The SFA was proximally patent but diminutive and interrupted at Hunter’s canal. Due to the absence of aneurysmal degeneration and ischemic symptoms, the case was conservatively managed with a single anti-platelet agent, timed analgesics and instructions to avoid prolonged seated positions.

### 2.4. Case 4

**Figure 5:**
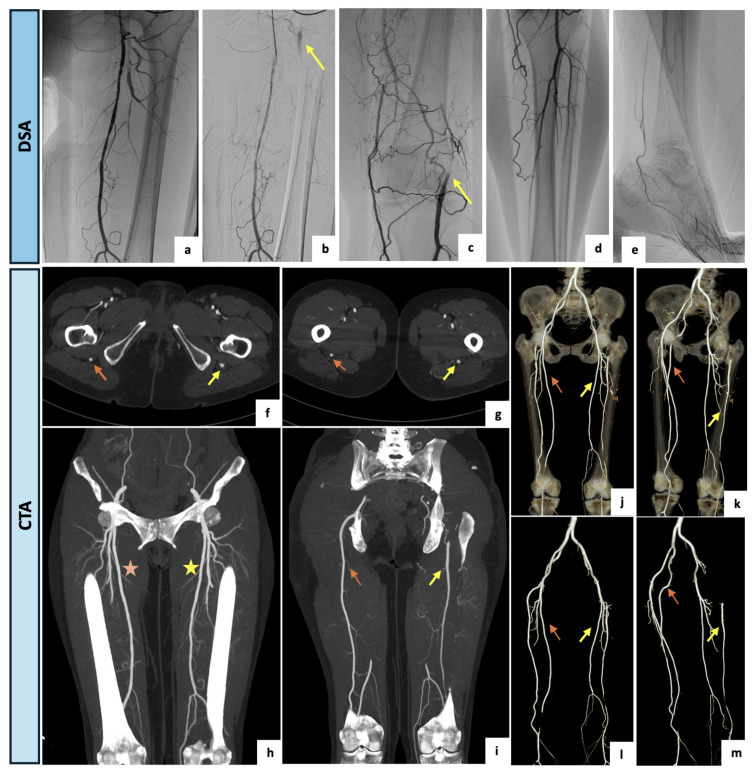
(**a**–**e**) Intraprocedural selective angiographic images obtained after a left antegrade common femoral access. (**a**–**e**) The angiograms demonstrate a diffusely hypoplasic and interrupted left SFA, lacking the usual continuation, and a popliteal artery supplied by collateral pathways originating from the distal small branches of the SFA and the deep femoral axis. (**b**,**c**) Surprisingly, an arterial vessel suggestive of incomplete PSA was retrograde perfused via deep femoral collateral pathways and visualized both proximally and distally as brief tracts (yellow arrows). (**f**–**i**) Axial and coronal maximal intensity projection views of the CTA confirming a bilateral PSA. On the left side, a type 2A PSA (yellow arrows) and a distal significant stenosis of the SFA (yellow asterisk) were detected. The right lower limb presented a type 1 complete PSA (orange arrow) along with a regular SFA (orange asterisk). (**j**–**m**) Three-dimensional reconstructions with and without bone structures. On the left side, a type 2a PSA with a proximal focal occlusion and re-habitation via gluteal collateral pathways, and a distal femoral significant stenosis was detected (yellow arrows). The right lower limb presented a type 1 complete PSA (orange arrows).

A 61-year-old woman was referred to the outpatient clinic of the Vascular Surgery Department for a type IIb bilateral claudication of her lower extremities with a recent worsening on the left side. The patient had a clinical history of active cigarette smoking, arterial hypertension, type 2 diabetes and moderate-to-severe renal impairment (eGFR of 29.9 mL/min/1.73 m^2^). Right peripheral pulses were present whereas on the left side the femoral pulse was diminished, and distal pulses were not palpable. Perfusion, sensation and motor function were bilaterally maintained. The arterial DUS of the lower limbs showed a complete occlusion of the left SFA at the Hunter’s canal with a restoration of blood flow at the level of the articular tract of the popliteal artery, where a monophasic and low peak systolic velocity flow was detected. On the right lower limb, physiological popliteal and tibial flow were assessed. After a multidisciplinary discussion of the clinical case, a primary angiographic approach with attempted left SFA revascularization was indicated with the aim of reducing the contrast medium burden. After an adequate periprocedural hydration protocol and under local anesthesia, a left antegrade common femoral access was obtained. The preliminary selective digital subtraction angiography showed a hypoplasic and interrupted left SFA, lacking the usual continuation. The popliteal artery was instead supplied by collateral pathways originating from distal small branches of the SFA and the deep femoral axis. Surprisingly, an arterial vessel suggestive of incomplete PSA was retrograde perfused via deep femoral collateral pathways and visualized both proximally and distally as brief tracts. Due to the incidental finding of the PSA, the case was rediscussed, and CTA was accordingly indicated. Upon CTA examination (Figure 5), a bilateral PSA was found. On the left side, a type 2A PSA with a proximal focal occlusion and re-habitation via gluteal collateral pathways, as well as a distal femoral significant stenosis, were detected. The right lower limb presented a type 1 complete PSA. The patient was thus submitted to a medical management program consisting of smoking cessation, exercise training, glucose-lowering therapy, anti-platelet agents and cilostazol, with an initial slight improvement of symptoms. Since the PSA assumed a dominant role as the main blood supplier to the left lower limb, the patient qualifies as a candidate for endovascular recanalization with PSA angioplasty if symptoms worsen or persist.

## 3. Discussion

Although PSA is a rare vascular anomaly, its presence is in most of cases of clinical relevance. As reported in the existing literature, 31–63% of cases of PSA occur with lower limb ischemia and up to 25% with ischemia at a critical stage [15,23,24]. Furthermore, the high rates of thrombo-embolic and aneurysmal complications make PSA a limb-threatening condition, leading to amputation in 8–10% of patients, even at an advanced age [8]. Nevertheless, PSA can be diagnosed based on a physical examination with the detection of leg ischemia, a history of calf claudication, the presence of a pulsatile gluteal mass or the pathognomonic Cowie’s sign; the coexistence of sciatic nerve compression symptoms mimicking sciatica make the diagnosis challenging and often retrospective after radiological imaging [12,23]. In the four reported scenarios, patient age, lifestyle factors, medical history and clinical presentations were extremely different and unspecific. Computed tomography angiography was the decisive diagnostic modality in all four cases (Table 1). In case 4, the initially avoided CTA was nonetheless crucial to further investigate the angiographic findings and to diagnose the contralateral PSA. The advantages of CTA in the diagnostic-therapeutic process for PSA are several and significant. Firstly, CTA can provide information about PSA and SFA anatomical types and arterial courses, and a complete evaluation of distal peripheral arteries [8,12]. Secondly, the key role of a CTA examination as a diagnostic tool in PAD has been largely recognized due to its safety, availability, high spatial and contrast resolution and rapid acquisition protocols [14]. Primarily in the femoral–popliteal arterial district, CTA has demonstrated high diagnostic accuracy (an overall sensitivity of 95–97% and a specificity 91–98%) in the evaluation of hemodynamically significant stenoses [13]. CTA is also capable of determining the presence, size and location of a PSA aneurysm. CTA has the advantages of evaluating an aneurysm’s relations with the surrounding structures, the presence of bony landmarks, the amount of intramural thrombus and the possible signs of distal embolization or previous rupture [25]. CTA can also diagnose completely occluded PSAs not able to be visualized by conventional angiography. Lastly, a CTA examination is an optimal panoramic imaging modality for pre-treatment (surgical, endovascular or hybrid) planning and for post-treatment evaluation [25]. Despite CTA being commonly preferred due to its widespread availability and the rapid speed of acquisition, magnetic resonance angiography (MRA) could be employed in cases of severe allergy to iodinated contrast or chronic renal failure, maintaining a comparable diagnostic accuracy [26]. Treatment was traditionally reserved for symptomatic cases of PSA while continued surveillance, usually with a DUS, was recommended for asymptomatic cases [3,5,8]. The management of PSA is determined by several factors, mainly consisting of the anatomical variations of PSA, the vascular anatomy of the ilio-femoral system, the severity of symptoms, the presence of aneurysmal changes and their characteristics [20,23]. In type 1 PSA, treatment is required only in case of aneurysmal formation or thromboembolic complications. Aneurysm exclusion can be performed by surgical ligation or endovascular techniques, including coiling, vascular plugs or stent-graft placement [12]. In type 2 PSA, revascularization is needed due to its leading role in the limb’s blood supply. Therefore, the management of PSA stenoses or occlusions should follow a patient’s clinical status and the lesions’ characteristics [26]. Surgical ilio-popliteal or femoro-popliteal bypass has so far been considered to be the preferred approach [8,20]. Its employment is, currently, reserved for long occlusions due to its high invasiveness, longer hospitalizations and the need for general anesthesia. The great majority of existing studies regarding PSA consist of single case reports or small series, whereas large series and multicentric studies are still missing, with the review of 40 patients by Koike et al. being one of the largest [9]. Furthermore, the available data on treatment outcomes, complications and long-term follow-up arise from studies involving PSA aneurysms or severe ischemic complications that were mostly treated by endovascular or hybrid approaches [20,23]. Unfortunately, studies comparing different treatment strategies (medical, endovascular, surgical and hybrid) and long-term outcomes of conservatively managed cases are lacking. However, favorable procedural outcomes, high rates of technical and clinical success, a low risk of complications and good results in terms of primary and secondary patency, symptoms recurrence and aneurysmal regression were reported in those submitted to endovascular treatment [9,23,24]. In the latest reviews, the use of the less invasive endovascular approaches for symptomatic PSA short steno-occlusions was also positively reported [15,27]. Nevertheless, further studies are needed to evaluate long-term outcomes of all the endovascular techniques, to optimize selection criteria and to evaluate overall complication rates, including stent-graft fracture, thrombosis, restenosis and aneurysm reperfusion.

## 4. Conclusions

PSA is an infrequent developmental anomaly with a high rate of aneurysmal and ischemic complications and a significant risk of amputation. CTA of the lower extremities is necessary for prompt identification of PSA and accurate treatment planning. In vascular anomalies, such as PSA, the use of a classification within the radiology report shared within a multidisciplinary team may be helpful in facilitating communication between different specialists and ultimately in managing the patient. Treatment strategies should be based on clinical and anatomical factors and selected after a meticulous analysis of the potential risks and benefits of the different available techniques. The different approaches (medical, endovascular, surgical and hybrid) should be discussed and evaluated on the basis of accurate imaging modalities (CTA or MRA), clinical symptoms and patient conditions. The present series has also confirmed the key role of a multidisciplinary approach to PSA involving diagnostic radiologists, interventional radiologists and vascular surgeons in the decision-making process regarding the treatment of PSA.

## Figures and Tables

**Table 1:** Summary table listing the main characteristics of the included cases.

Case	Age	Sex	Clinical Presentation	Imaging Modality	Type of PSA	Management	Follow-Up (FU) Results
1	79	Female	Right flank and buttock pain, burning sensation in gluteal region and in the back of right lower limb	CTA	Right limb: Type 1 with aneurysmatic PSA Left limb: Type 2a	Embolization with “sandwich technique” of right aneurysmatic PSA.	Clinical FU: complete disappearance of symptoms, no required pain medication. CTA FU (1-month): complete exclusion of PSA aneurysm.
2	53	Male	Critical ischemia with rest pain of the right lower limb	CTA	Right limb: Type 2a with aneurysmatic PSA	Hybrid approach: right femoral-tibial bypass and endovascular embolization of the aneurysmal PSA.	Clinical FU: symptoms improvement. CTA FU (3-month): patent bypass, completely excluded aneurysm.
3	81	Female	Moderate and recurrent back pain occasionally requiring pain medication	CTA	Right limb: Type 2a	Conservative management: single anti-platelet agent, analgesics.	Clinical FU: symptoms improvement with conservative therapy. No CTA FU needed.
4	61	Female	Type IIb bilateral claudication with left side worsening	CTA	Right limb: Type 1 Left limb: Type 2a	Medical management: smoke cessation, exercise training, glucose-lowering therapy, anti-platelet agents and cilostazol.	Clinical FU: progressive improvement.

## Data Availability

All data of the current study are available from the corresponding author upon reasonable request.
